# Bitmap and vectorial hologram recording by using femtosecond laser pulses

**DOI:** 10.1038/s41598-021-95665-5

**Published:** 2021-08-12

**Authors:** Y. Kotsiuba, I. Hevko, S. Bellucci, I. Gnilitskyi

**Affiliations:** 1 NoviNano Lab LLC, Lviv, Ukraine; 2 Karpenko Physico-Mechanical Institute of the NAS of Ukraine, Lviv, Ukraine; 3 INFN-Laboratori Nazionali di Frascati, Via E. Fermi 54, 00044 Frascati, Italy; 4 Department of Photonics, Lviv Polytechnic National University, Lviv, Ukraine

**Keywords:** Applied optics, Optical techniques

## Abstract

In this paper, we present two approaches for recording a quasi-hologram on the steel surface by femtosecond laser pulses. The recording process is done by rotating the polarization of the laser beam by a half-wave plate or a spatial light modulator (SLM), so we can control the spatial orientation of the formed laser-induced periodic surface structures (LIPSS). Two different approaches are shown, which use vector and bitmap images to record the hologram. For the first time to our knowledge, we managed to record a hologram of a bitmap image by continuously adjusting the laser beam polarization by SLM during scanning. The developed method can substantially improve hologram recording technology by eliminating complex processing procedures, which can lead to increasing the fabrication speed and reducing the cost.

## Introduction

In recent years, there has been a growing demand for optical elements designed for creating three-dimensional images and visual effects, a new level of information security, high-precision control and manipulation of the laser radiation as well as high-capability memory storage systems. Holography makes it possible to fully implement all these tasks^1^. In classical holography, various photosensitive media are used to register holograms: silver halides, dichromated gelatin, photopolymers, photothermoplastics, etc.^2^. The digital holography substantially expands the capabilities of traditional holography, since it does not require a laser to record hologram and can be used to visualize multidimensional information^3–5^. It allows the design of unique three-dimensional amplitude and phase fields that can expand the variety of holographic elements. Today, it is known about its application in microscopy^6^, quantitative phase imaging^7^, tracking of particles motion^8^, measurement of gases or liquids flow in three-dimensional space^9^, volume visualization of biological objects^10^, information encryption^11,12^. However, with all the advantages of numerical methods, the recording time of single hologram is quite long, and the process itself is much complicated since some requirements should be met. For instance, ensuring the linearity of recording, and the absence of unwanted light or vibrations, that can blur holographic fringes.

The ultrashort laser pulse can provide an alternative method for recording a hologram^13^ since the pattern of obtained structures is similar to holographic fringes. Direct laser processing methods, compared to the traditional ones, excludes many expensive and complex technological processes, like chemical processing or high vacuum. Processing the material with a femtosecond laser pulse also yields benefits in terms of speed and cost. Using modern laser system in combination with high-precision galvoscanners allows one to achieve high processing speed while, at the same time, providing the good quality of the nano-structures, so-called laser-induced periodic surface structures (LIPSS). LIPSS were firstly demonstrated back in 1965 by Birnbaum^14^. LIPSS are self-organization phenomena which can be created upon linearly polarized ultrashort laser pulses. The most common model of LIPSS formation supposes the generation of the nanostructures due to the interference of Surface Electromagnetic Wave (SEW) with the incident laser wave exciting a coherent surface plasmon-polariton (SPP) wave^15^. The LIPSS are obtained on various materials irradiated over a broad range of laser wavelengths and pulse durations, at high speeds and on large areas^16,17^. So, Gnilitsky et al.^18^ reported the formation of LIPSS at a speed above 1 cm^2^/s. The high processing speed combined with the simplicity of the technology reduces its cost. Taking into account the operating costs of the femtosecond laser and positioning system, the estimated cost of this technology is 0.1 €/cm^2^^19^. Today nanostructure patterning is possible on the surface of metals^20,21^, semiconductors^22^, glass^23^ and polymers^24^. Such processing allowed producing hydrophobic surfaces^25^, colourization effect^26^, improving of the tribological characteristics of parts^19^, realization of antibacterial surfaces^27^, etc.

The recording holograms with an ultrashort laser pulse can be achieved only with control of the LIPSS spatial parameters, which is still difficult to achieve. Presently, it is known that there exists the possibility to adjust the spatial orientation of LIPSS by rotating the polarization of laser beam, control their spatial frequency by changing the angle of incidence and depth of the structures by tuning the energy density. In^28^ authors report about the recording of a holographic image on a metal surface by manipulating the orientation of nanostructures with a half-wave plate. Other authors suggested SLM^29^ or a liquid crystal polarizer^30^ for generating LIPSS with different orientation. Possible applications could find patterns described in^31^. Here, using a couple of cross-polarized femtosecond pulses, Liu et al. achieved the formation of structures perpendicular to the scanning direction. Using direct laser interference patterning (DLIP) Voisiat et al.^32^ managed to avoid colour variation in the recorded nanostructured image for an arbitrary viewing angle. Another example of recording hologram using DLIP is described in^33^.

In this paper, we introduce two different methods of recording quasi-holograms on the surface of steel by varying the spatial orientation of LIPSS. The proposed methods use a half-wave plate and SLM to record a hologram of a vector and a bitmap image.

## Results and discussion

In this section, we present the results of recording quasi-holograms of vector and raster images by two different methods: by rotation of the polarization with half-wave plate and spatial light modulator. The obtained structures with quantitative quality analysis are shown.

### Recording holograms of vector image

**Figure 1:**
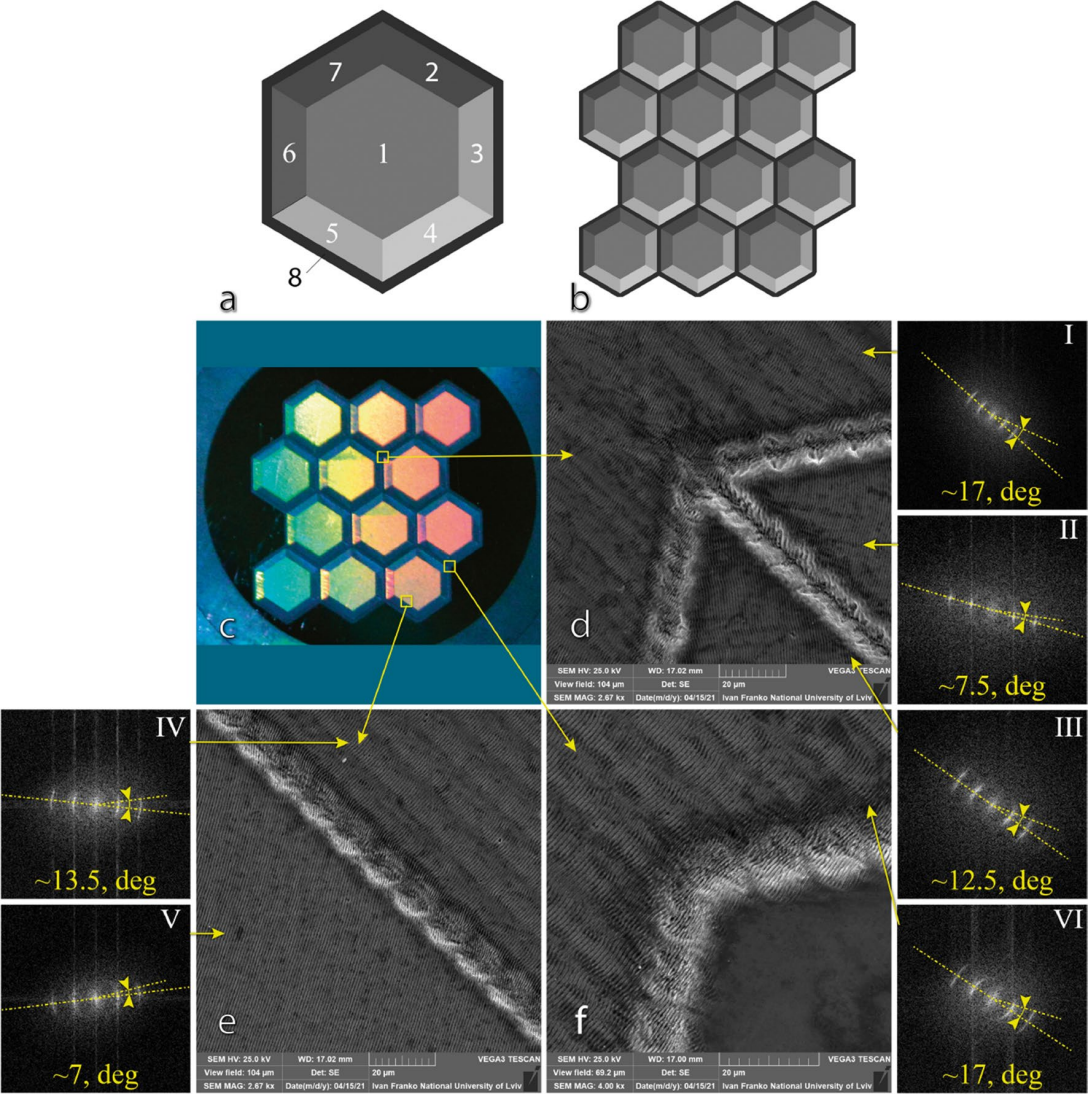
Input vector image (**a**) and SEM-image of different segments of the hologram with Fourier transforms and determination of the approximate angle of orientation of nanostructures (**b**) Recorded quasi-hologram (**c**) and SEM images of different parts of the hologram (**d**–**f**). Insets I–VI indicate dispersion LIPSS orientation angle (DLOA) for various segments of quasihologram.

For hologram of the vector image, we chose wavelength *λ* = 1030 nm taking the pulse duration *τ* = 266 fs, the pulse repetition rate *υ* = 500 kHz, the scanning speed *V* = 0.8 m/s, scanning step *d* = 5 µm and the pulse energy *E* = 0.9 µJ. The fluence value was 1.04 J/cm^2^. For recording, we used the vector image shown in Fig. 1a. The hologram recorded on the steel surface is shown in Fig. 1b.

All the hexagons of the input image are divided into 8 segments (as is shown in Fig. 1a). After each sequential scan, we changed the polarization of the laser beam, providing a unique spatial orientation of LIPSS within the scanned segment. The polarization was tuned by rotating the half-wave plate taking the range from 0° to 90° with a step 11.25°. The obtained quasi-hologram and SEM images of the resulting structures are shown in Fig. 1c–f.

The quality of the LIPSS was identified by a method named Dispersion of LIPSS orientation angle (DLOA) that was described in ^18^. DLOA was measured at different segments of the hexagon. In Fig. 1d, DLOA of segment depicted by inset (II) is equal to 7.5°, while insets (I) and (III) are measured to be 17° and 12.5° respectively. Figure 1e shows DLOA on one segment 13.5° (inset IV) while for the other one—7° (inset V). Figure 1f displays DLOA on segment that is equal to 17° (inset VI). Segments with DLOA 7.5° and 7° are according to the method covered with highly-regular LIPSS (HR-LIPSS). The segments with means of DLOA from 12.5° to 17° are textured with LIPSS of good quality with some distortions and small bifurcations. Such differences in LIPSS quality can be explained by the impact on the LIPSS regularity of the scanning direction, with respect to the direction of the polarization. In case, the scanning direction is in line (and up to 45°) with polarization, the quality of LIPSS will be perfect. While, if the scanning direction is perpendicular to, the LIPSS quality will be strongly degraded, that is in good accordance with^18,34^.

The main advantage of this approach is the high recording speed and high quality of the obtained structures. By using a high-speed optical shutter, one can improve the method to record more complex vector images.

### Recording holograms of bitmap image

**Figure 2:**
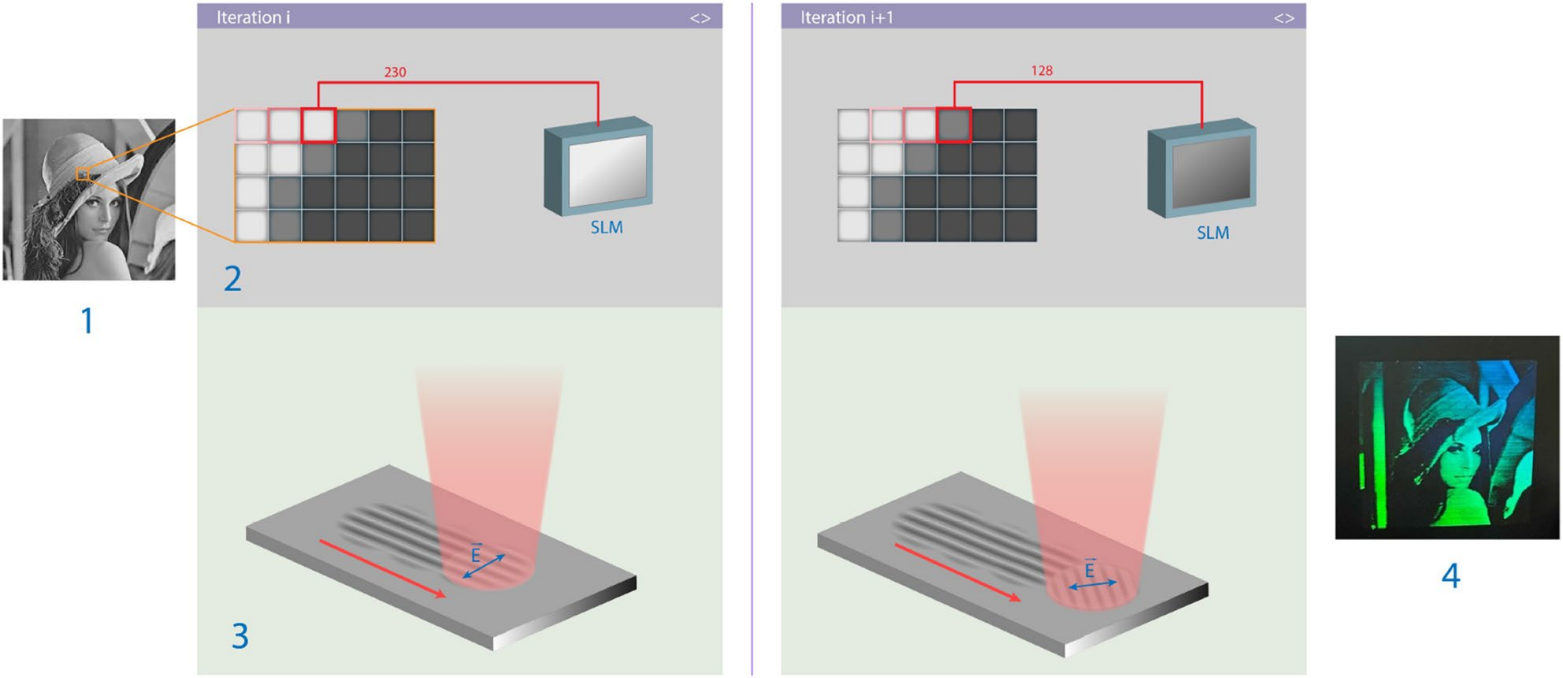
Procedure of hologram recording with ultrashort laser pulses: input image (1); sending a signal to the SLM (2) scanning the surface with a laser beam with the formation of LIPSS (3); recorded hologram (4).

The new proposed method for recording holograms with SLM is described in Fig. 2.

First, the input image (1) is converted to a grayscale color model with adjusting graystone range according to the linear range of the SLM. During laser scanning, the voltage of the whole SLM matrix is dependent on the pixel grey level (2) and switches with a certain period of time, providing control of the polarization of the incident beam, and the spatial orientation of LIPSS (3). By scanning row by row, we obtain a quasi-hologram of the bitmap image (4).

As can be seen from Fig. 2 recorded periodic structures have spatial orientation depending on the grey level value on the SLM. Those structures are quite similar to the diffraction grating with the period *d* and the dependence of the diffraction order as follows:

1 $$m \lambda = d(sin\theta_{m} - sin\theta_{in} \cdot cos\varphi )$$

where *m* denotes the diffraction order, *θ*_*m*_ and *θ*_*in*_ are the m-th order diffraction angle and incident angle respectively, and φ represents the angle between the grating vector and the light incident vector in the grating plane.

It is evident that at *φ* = 90° the intensity of the diffracted light of the m-th order reaches its maximum and sinusoidally decreases to the minimum at *φ* = 0°. In this work, we distinguish three brightness zones dependent on the azimuthal angle: within the lowest zone *φ* < 25°, while the highest brightness zone has φ > 65°, and all other values stand for the intermediate one.

The resolution of obtained patterns has an inverse dependence on the scanning speed with a constant SLM repetition rate. In our case, the spatial resolution was 0.05 mm^−1^ (200 dots per line for an area of 10 × 10 mm), which is a bit less than the theoretical minimum of 0.42 mm^−1^. There are two ways to increase it: by decreasing the recording speed or by using SLM with a higher repetition rate. The first way is definitely simpler but causes the recording time to increase. As for modern spatial light modulators, there are widely used models with a frame rate of 120 Hz, which is twice more than in our device. The most expensive high-speed models today have a frame rate up to 425 Hz.

**Figure 3:**
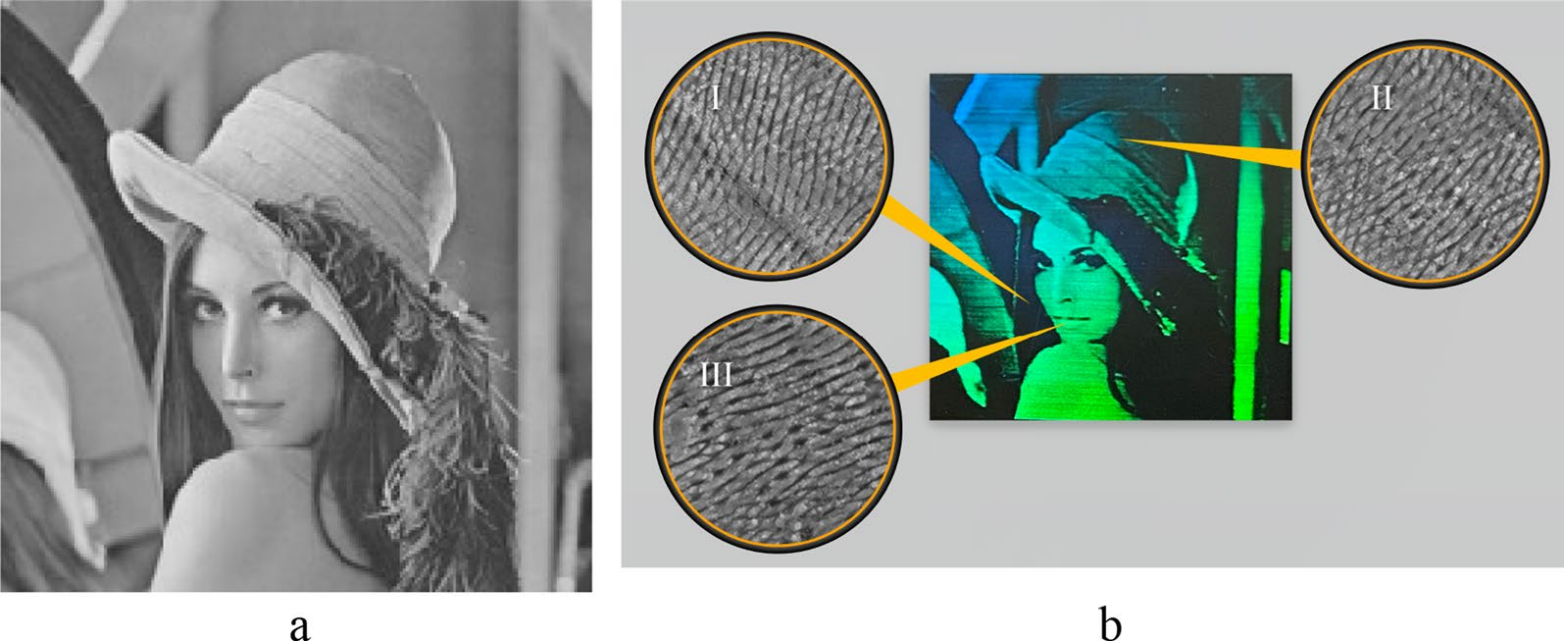
A bitmap image used for recording quasi-hologram (**a**). Recorded hologram with highlighted areas of low (I), intermediate (II) and high brightness level (III) (**b**).

In this approach, we should take into account two issues: the small frame rate of the SLM and the spectral working range. Hence, we chose *λ* = 515 nm with *τ* = 266 fs, and scanning speed *V* = 2.5 mm/s. The pulse repetition rate was *υ* = 16.7 kHz with a scanning step *d* = 5 µm and pulse energy *E* = 6.7 µJ. We chose the fluence value was 13.336 J/cm^2^. As an input, we used the image with a dimension of 200 × 200 (see Fig. 3a). The quasi-hologram with a size 10 × 10 mm recorded on the steel surface is shown in Fig. 3 (b). SEM images corresponding to the different brightness levels are also shown (Fig. 3I–III).

**Figure 4:**
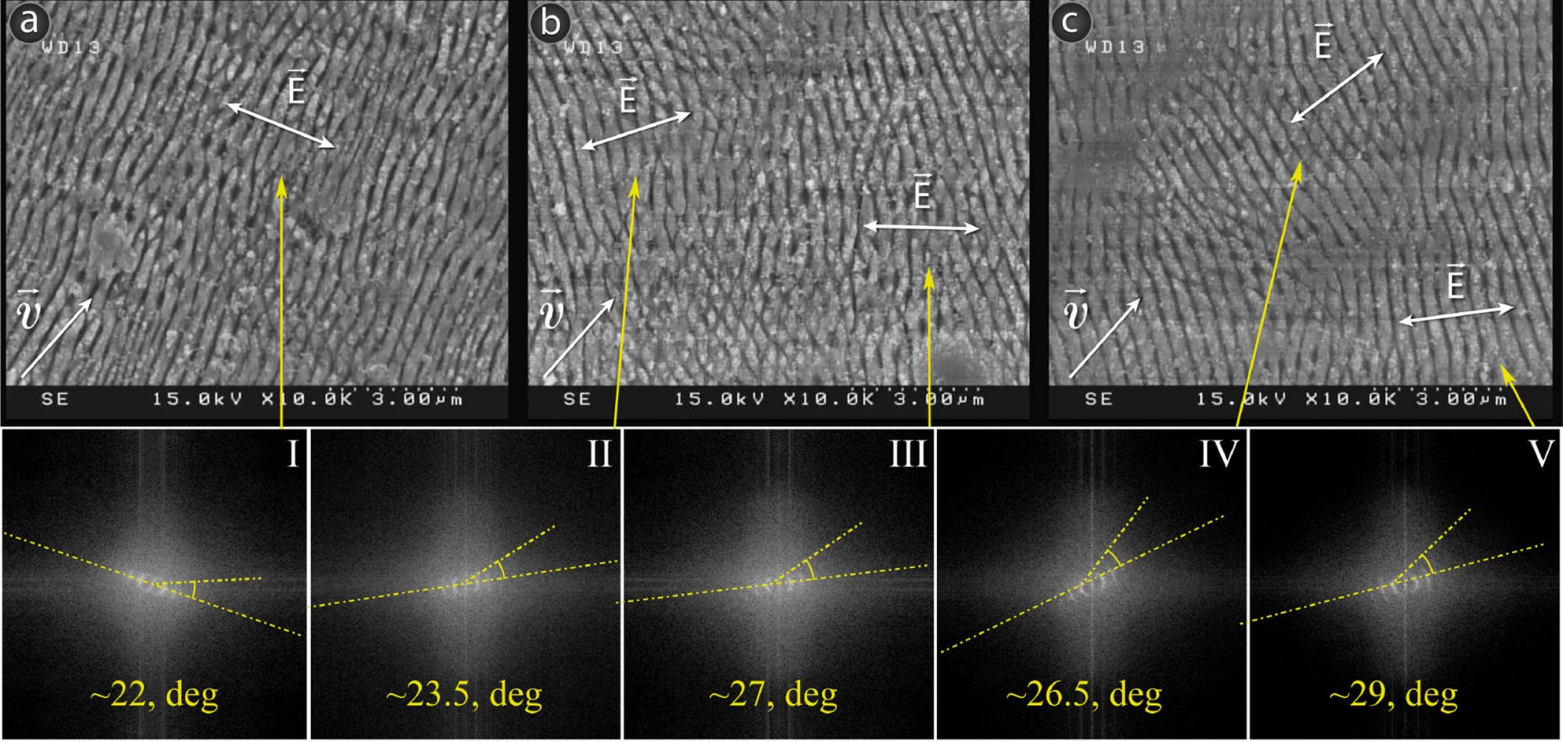
SEM images of different parts of the hologram: (**a**) high brightness; (**b**) intermediate level of brightness; (**c**) low brightness. Insets I–VI denote DLOA for various segments of quasihologram.

Taking into account the parameter "value" of the HSV color model, we can distinguish three main zones corresponding to different orientations of LIPSS: low brightness zone has V in the range 40–100, corresponding to structures of type (a) (see Fig. 2b); in the intermediate brightness zone V varies from 120 to 200 and corresponds to structures type (b); for the brightest zone V is in the range of 220–250 for structures of type (c). SEM images of different zones of the hologram are shown in Fig. 4.

From Fig. 4 one can see how the orientation of the LIPPS changes during dynamical rotation of the incident laser beam polarization. The SLM matrix enables relatively rapid switching of polarization vector compared to standard approaches, which use rotation of the half-wave plate. This allows continuous recording of single image with high resolution. However, to provide this for a constant area and SLM refreshing rate we must reduce the recording speed. DLOA of segments on Fig. 4a–c depicted by insets I–V is equal to 22°–29° that reveal LIPSS of low regularity. These results suggest a possible distortion between laser source and SLM during their synchronization. However, we have demonstrated on Fig. 4 the possibility of obtaining non-symmetrical LIPSS, which can find a potential application in obtaining non-symmetrical quasicrystals^35^.

## Materials and methods

### Experimental setup

**Figure 5:**
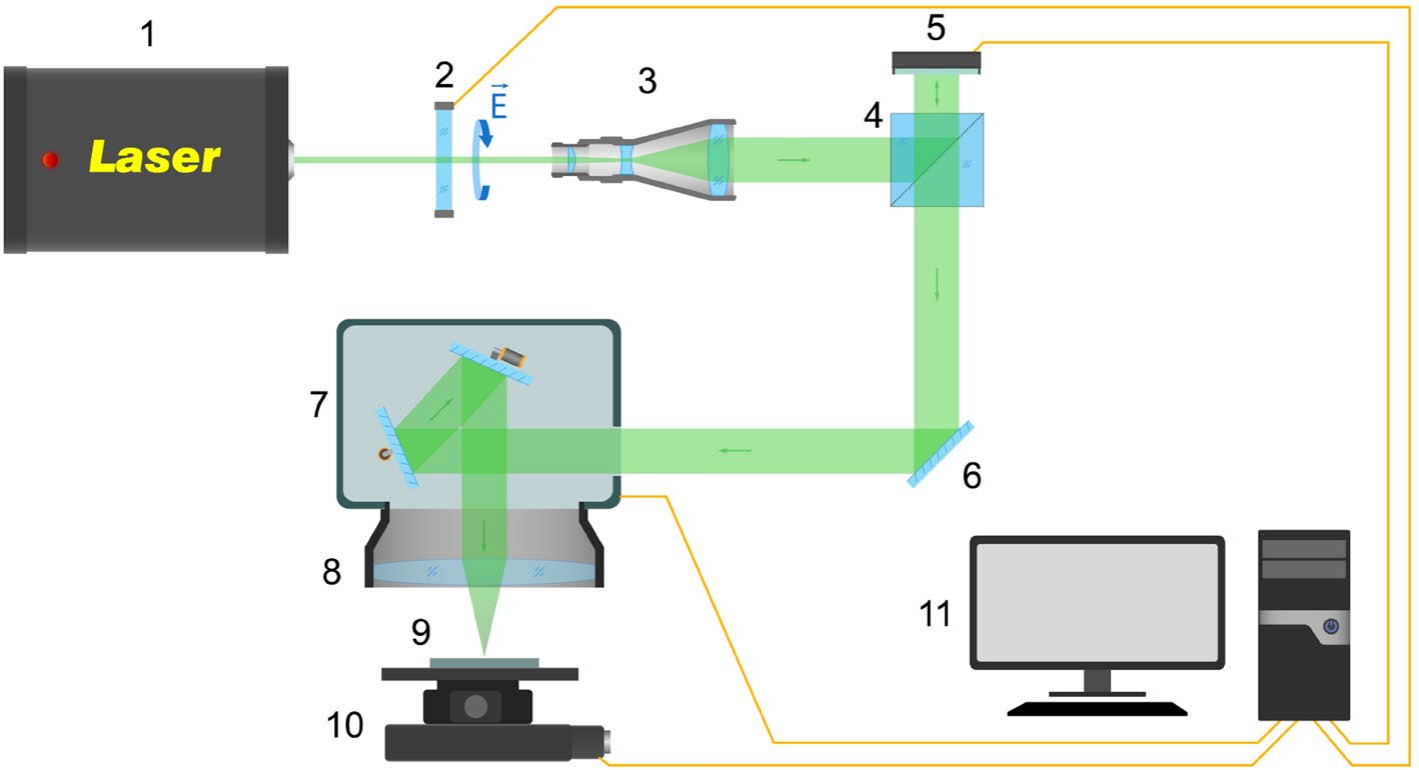
Optical scheme of hologram recording: laser (1), half-wave plate (2), beam expander (3), beam-splitting cube (4), spatial light modulator (5), mirror (6), galvo scanner (7), F- Theta lens (8), sample (9), six- axis positioner (10), PC (11).

Hologram recording was done on an optical setup schematically shown in Fig. 5, which includes galvo scanner, a spatial light modulator, and a half-wave plate.

The light beam from the Pharos laser system (1) passes through the dynamic beam expander (3) to cover the whole area of the SLM matrix (5). By adjusting the polarization of the incident light with a half-wave plate (2) we achieve the amplitude modulation regime of the spatial light modulator. The expanded beam propagates to the light-splitting cube (4) and reflects from the SLM matrix. The reflected beam travels through the system of mirrors (6) and enters the galvo scanner (7), which uses an F-Theta lens and (8) to scan the sample (9). With a six-axis positioner (10) we provide focusing during sample processing. The control of all devices performed using a PC (11).

The setup allows implementing two approaches for recording quasi-holograms on the different substrates. The first method requires static polarization of a laser beam. Separate parts of the holographic image are recorded sequentially, gradually changing the polarization with the half-wave plate after scan. The second approach involves a dynamic change of the laser beam polarization using SLM during a single scan. The software developed by the authors provides control and synchronization of all devices. The described means allow recording quasi-holograms using both bitmap and vector images. A detailed description of all recording approaches is discussed below.

### Method of recording holograms of vector images

The easiest method to implement and the least time-consuming one is that with static beam polarisation, which is suitable for segmented vector images. The hologram is recorded by scanning a substrate with different light polarization acquiring a different spatial orientation of LIPSS for each segment of the image. An example of a vector image used to record a hologram in this work is shown in Fig. 1b.

By modifying the LIPSS orientation for each segment of the hexagon one can achieve the pseudo volume effect. More complex images require reducing the size of individual segments. Hence, when processing at high speed and pulse repetition rate one cannot avoid parasitic transition lines between segments. Recording a high-quality hologram in a relatively short period of time requires a high-speed shutter, such as an acousto-optic modulator, with a switching rate of 2–10 μs^36^.

### Method of recording holograms of bitmap images

Another approach was used to record the hologram using a bitmap image. The main difference is that the processing is carried out continuously. The polarization of the incident laser beam is switched dynamically by the spatial light modulator during the scanning. Thus, one can obtain different spatial orientations of LIPSS by a single scan. This approach does not require high-speed shutters to prevent parasitic lines. The main drawback is the slow processing speed, due to the low frame rate of the SLM. For recording a single image row, the signal in the SLM should switch M times for the image with dimension M × N. When recording a hologram with a size of 10 × 10 mm and dimension 500 × 500 pixels, the maximum possible processing time can be 10 mm/s at a frame rate 500 Hz.

**Figure 6:**
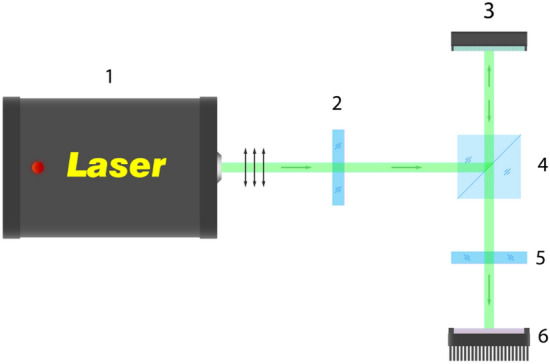
Optical scheme for studying the dependence of the rotation of the polarization angle on the grayscale level at the SLM input: (1) a laser with linearly polarized light; (2) half-wave plate; (3) LCoS SLM; (4) beam-splitting cube; (5) analyzer; (6) laser power meter.

Since the spatial orientation of LIPSS is controlled by SLM, it is necessary to determine the dependence of the polarization angle variation on the grayscale level. In this work, the SLM was the LCoS matrix, which we extracted from the budget projector LG PH 150G with a frame rate of 59 Hz. In order to study the appropriate dependence, we used a simple optical scheme with a half-wave plate and an analyzer (see Fig. 6).

The total transmission of this scheme is equal to:

2 $$T = \cos^{2} \theta - \sin^{2} \varphi \sin^{2} \left( {\frac{\delta }{2}} \right)$$

where *θ* denotes the angle between the laser polarization after the half-wave plate and the analyzer, *φ* represents the angle between the polarization and the optical axis of the liquid crystal, δ stands for polarization retardation related to voltage applied.

Obviously, when *θ* = 90° and *φ* = 45° the transmission is equal to:

3 $$T^{\prime} = \sin^{2} \left( {\frac{\delta }{2}} \right)$$

Having obtained the experimental dependence of the intensity variation at each grayscale level and knowing the input intensity, we calculated the dependence of the polarization angle rotation for our LcOS matrix.

**Figure 7:**
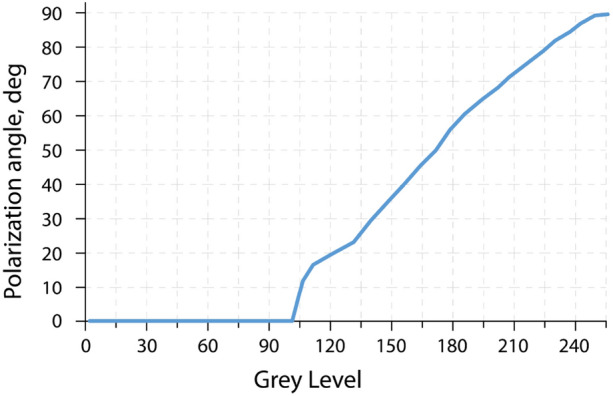
Dependence of the polarization angle variation on the grayscale on the SLM.

As can be seen from Fig. 7 the obtained dependence is quite nonlinear. Within the range from 0 to 100 variation of the polarization angle is not being observed. For our purpose, optimal is a linear area in the range of 130–255. Obviously, the image greyscale level range must be adjusted before recording according to the operating range.

## Conclusion

Two different approaches for recording the quasi-hologram with the ultrashort laser pulse are described and the obtained holograms are shown. The periodic structures of the obtained holograms provide good coloring even upon a second approach with a cheap SLM. At this moment, we are expanding the potential application of our methods by looking for the possibility of recording hologram on other materials. Mostly, attention is being paid to transparent materials, such as glass or transparent polymers. The obtained results will be the basis for a new technology of recording diffraction optical elements or holograms by ultrashort laser pulses. This technology can find an application for producing a whole variety of holographic optical elements, such as lenses, optical multipliers, spatial filters, compensators, or even holographic memory systems. Other possible applications of recorded quasi-holograms consist in creating unique optical effects and rainbow holograms of 2D and 3D objects for light art decoration, and advanced counterfeit protection.
